# Correction: Impact of different blood pressure targets on cerebral hemodynamics in septic shock: A prospective pilot study protocol—SEPSIS-BRAIN

**DOI:** 10.1371/journal.pone.0350851

**Published:** 2026-06-02

**Authors:** Pedro Cury, Rogério da Hora Passos, Fernanda Alves, Sérgio Brasil, Gustavo Frigieri, Fabio S. Taccone, Ronney B. Panerai, Juliana Caldas

The Competing Interests statement for this article is incorrect. The correct Competing Interests statement is as follows: The authors have read the journal’s policy and have the following competing interests: SB is a senior advisor for Brain4Care. GF is the Brain4Care founder. This does not alter our adherence to PLOS ONE policies on sharing data and materials. There are no patents, products in development or marketed products associated with this research to declare.
