# Supplementary figures and images for: Impact of different blood pressure targets on cerebral hemodynamics in septic shock: A prospective pilot study protocol—SEPSIS-BRAIN
Source: PLoS One. 2024 Oct 14;19(10):e0304412. doi: 10.1371/journal.pone.0304412 (PMC11472940; doi:10.1371/journal.pone.0304412)

**S1 Fig**

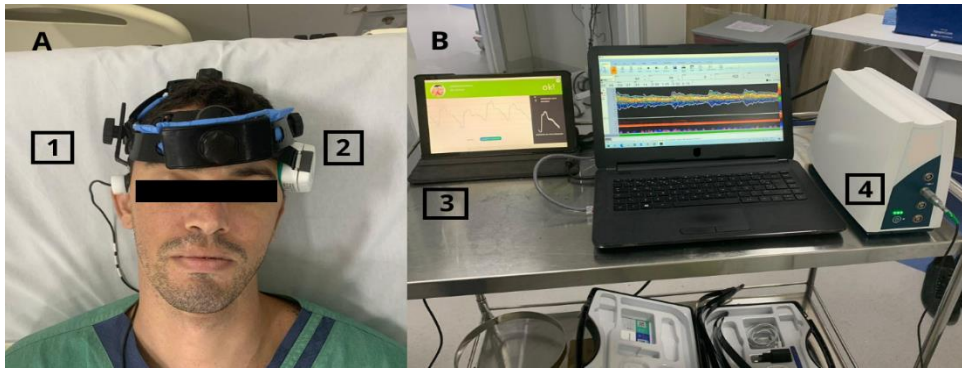

Supplement: S1 Fig — Cerebral monitoring for data collection of the study. 1) Transcranial Doppler probe maintained in position with a probe-holder; 2) Brain4care sensor; 3) Brain4care main display showing the cerebral compliance curve; 4) Transcranial Doppler device main display showing cerebral blood velocity of the middle cerebral artery. (Under a CC BY license, with permission from Dr. Thiago Passos. Vincenzo Lionetti, original copyright 2023). (PDF) [file pone.0304412.s001.pdf]

S2 Fig

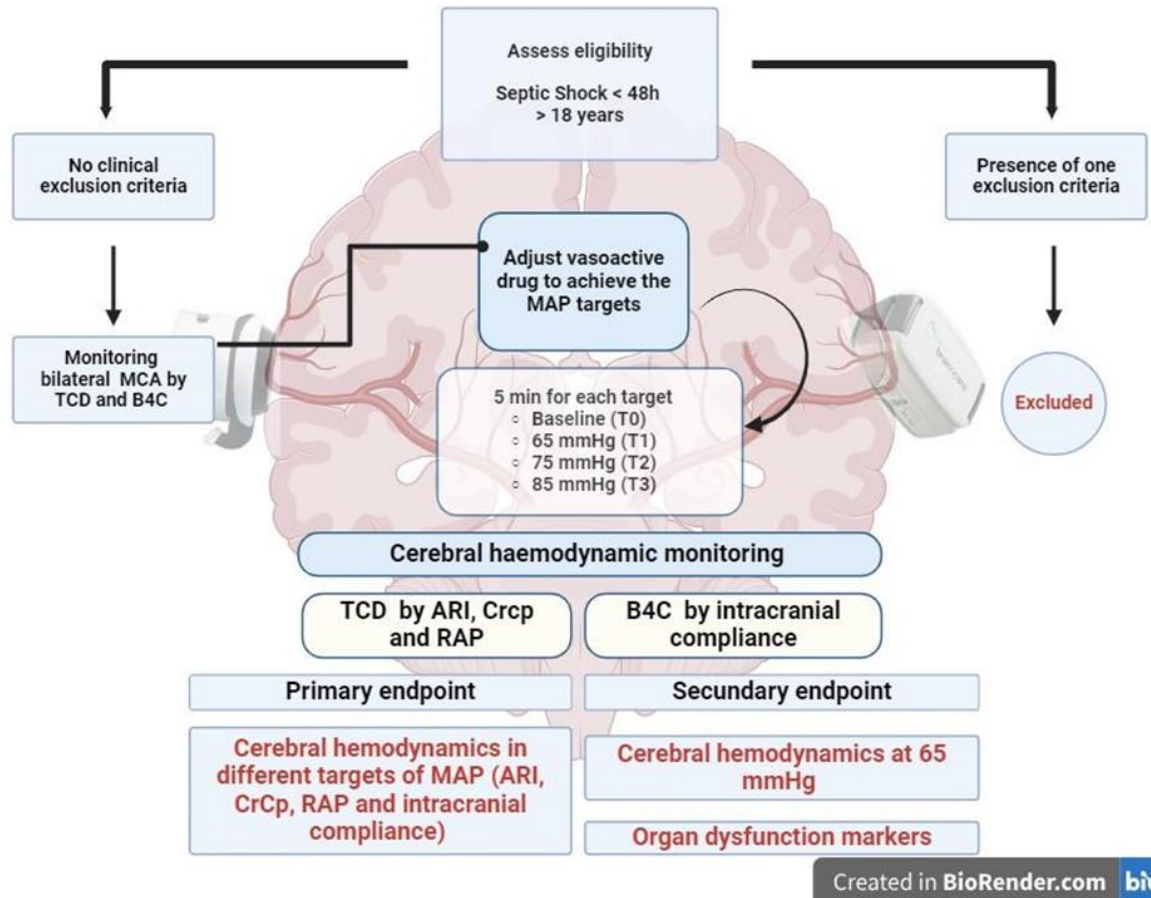

Supplement: S2 Fig — Flowchart: MCA, middle cerebral artery; TCD, transcranial Doppler; B4C, brain4care; ARI, autoregulation index; CA, cerebral autoregulation; Crcp, critical closing pressure; RAP, resistance-area product. (PDF) [file pone.0304412.s002.pdf]
