# Supplementary material for: Impact of different blood pressure targets on cerebral hemodynamics in septic shock: A prospective pilot study protocol—SEPSIS-BRAIN
Source: PLoS One. 2024 Oct 14;19(10):e0304412. doi: 10.1371/journal.pone.0304412 (PMC11472940; doi:10.1371/journal.pone.0304412)
Supplement: S1 Table — MAP: mean arterial pressure, MCA: middle cerebral artery, TCD: transcranial doppler, B4C: brain4care, ICU: intensive care unit, APACHE: Acute Physiologic and Chronic Health Evaluation, SOFA: Sequential Organ Failure Assessment, ARI: autoregulation index CrCP: critical closing pressure, RAP: resistance-area product. (PDF) [file pone.0304412.s003.pdf]

S1 Table

		STUDY PERIOD					
	**Recruitment**	**Allocation**	**Intervention protocol (5 min on each MAP target)**			**Clinical outcome**	
	07/23 – 06/25	***< 48 hours ICU admission***	***MAP 65 mmHg***	***MAP 75 mmHg***	***MAP 85 mmHg***	***7th day***	***28th day***
**ENROLMENT:**							
**Eligibility screen**	x						
**Informed consent**	x						
**Allocation**		x					
**INTERVENTIONS**							
Induce different MAP targets			x	x	x		
Monitor bilateral MCA by TCD and B4C			x	x	x		
ASSESSMENTS							
**Sex, age, time, time since admission to the ICU, infection site, comorbidities, APACHE, SOFA, vasopressor dosage, blood analysis**		x					
**TCD: ARI, CrCP, RAP**			X	X	X		
**B4C: P2/P1 ratio**			X	X	X		
**Brain dysfunction**		X				x	x
**Ventilation free days**							x
