# Supplementary material for: Impact of different blood pressure targets on cerebral hemodynamics in septic shock: A prospective pilot study protocol—SEPSIS-BRAIN
Source: PLoS One. 2024 Oct 14;19(10):e0304412. doi: 10.1371/journal.pone.0304412 (PMC11472940; doi:10.1371/journal.pone.0304412)
Supplement: S2 Table — * Ischemic or hemorrhagic stroke, aneurysm, arteriovenous malformation, hydrocephalus, neurological surgery, central nervous system infection. ** Reported or written in medical records. *** Partial pressure of arterial carbon dioxide (PaCO2) > 65mmHg. **** Intra-aortic balloon pump (IABP) and Extracorporeal membrane oxygenation (ECMO). (PDF) [file pone.0304412.s004.pdf]

S2 Table

Inclusion criteria	Exclusion criteria
• Age > 18<br>• Septic shock < 48h<br>- The timing will be counted from ICU admission or the start of noradrenaline infusion for patients already in critical care units.	• No temporal window for TCD<br>• Hepatic or uremic encephalopathy<br>• Pregnancy<br>• Acute or prior neurological insult*<br>• Exogenous intoxication<br>• Dementia**<br>• Severe hypercapnia***<br>• Instability for acute arrhythmia<br>• Extracorporeal support device by modifying the pulse waveform****<br>• Extreme severity, with imminent risk of death
