# Supplementary material for: Impact of different blood pressure targets on cerebral hemodynamics in septic shock: A prospective pilot study protocol—SEPSIS-BRAIN
Source: PLoS One. 2024 Oct 14;19(10):e0304412. doi: 10.1371/journal.pone.0304412 (PMC11472940; doi:10.1371/journal.pone.0304412)
Supplement: Attachment — Submitted filename: RESPONSE TO REVIEWERS.docx [file pone.0304412.s005.docx]

Dear Vincenzo Lionetti,

Thank you very much for conducting the review process for our manuscript PONE-D-23-22748 and for the opportunity to address the points raised by the reviewers. We found their comments relevant and these were taken into account to produce a revised version of the manuscript. We trust that you will find that our manuscript has been considerably improved, and we hope the revised version will meet your acceptance criteria. Our replies to each of the comments from the reviewers are given below where we also explain corresponding changes made to the manuscript.

**Reviewer #1:**

**Q1. ABSTRACT:** Please explain the method rather than using the wording ‘in a program capable of evaluating the autoregulatory index(ARI), CrCP and resistance-area product (RAP).

**Reply: Thank you for your careful reading of the manuscript and for your suggestions. We have implemented all your recommendations. Please, see lines 37 and 41.**

**Q2.** Avoid abbreviations and make the text as self-explanatory as possible. The Abstract should provide an adequate summary of the protocol without the need to consult the main body of text when indexed on search sites.

**Reply: We have now modified this point, thanks for your help with this.**

**Q3 PROTOCOL:**. Please explain why the data availability statements differ for this submission (Deidentified research data will be made publicly available when the study is completed and published) compared to the ClinicalTrials.gov registration (PLAN TO SHARE INDIVIDUAL PARTICIPANT DATA(IPD) No) or the dissemination policy text (data kept confidential for 5 years). Please provide an explicit statement for data availability that should include the location (eg. OSF, Zenodo, Figshare or similar).

**Reply – Many thanks for your suggestion. To address your concern, we have now modified the clinical trials.gov protocol and we will provide the data of the patients in the Zenodo.**

Q4. Please explain the difference in the SPRINT diagram which indicates study starts in July 2023 vs. ClinicalTrials(recruitment is ongoing, 7/7/2022) and the manuscript text (commencing 1/6/2023).

**Reply: We encountered some issues with our equipment and found it necessary to acquire new TCD probes. Consequently, our recruitment was interrupted for a period of several months. In response to this situation, we took the necessary step of updating the information on ClinicalTrials.**

Q5. Please indicate time periods for the different MAPs in the SPRINT diagram.

**R - Thank you. We have added the requested changes.**

**Q6.** From the manuscript: ‘We expect that these results will present a different pattern in patients with impaired cerebral autoregulation and altered intracranial compliance, and consider whether these parameters can be modified by different MAP levels, suggesting a personalized cerebral hemodynamics approach.’ Would it not be more correct tostate that different MAP can support CPP to a varying degree while the CA/ICC impairment is the same?

**Reply - The study aims to utilize different MAP targets (exposure) to assess CA and ICC (outcomes). We exclude subjects with neurological conditions (acute or chronic) and will describe variations in carbon dioxide, while keeping intracranial pressure constant. Assuming that other CA variables remain constant during the short data collection period, the modifying factor of CA indices would be MAP. The trial seeks to investigate whether manipulating pressure can modify CA in patients with septic shock. This is important because changes in cerebral microcirculation may occur even with normal systemic hemodynamics and could contribute to tissue hypoxia. Therefore, the first step will be to analyze the commonly targeted MAP of 65mmHg.**

Q7. From the manuscript ‘The primary aim is to assess cerebral hemodynamics (ARI, CrCP, RAP and P2/P1) in septic shock patients submitted to the 65 mmHg of MAP’. This statement needs to be elaborated. The dynamic autoregulation requires a variation in MAP and this is often reduced during a MAP directed therapy. What quality criteria will be used for the dynamic autoregulation? Will the time periods be changed if there is insufficient MAP variability to assess dynamic CVAR? A time period of five minutes seems insufficient to this reviewer. Can MAP be measured from several sites (eg. radial, brachial, femoral)? The ARI is derived from a step change in MAP, how will this be assessed at the baseline MAP 65 mmHg (only once increased to 75 mmHg)? What criteria will be used to define impaired dynamic autoregulation?

**Reply - This is an important point. Dynamic CA is the transient response of CBF to an acute change in BP, lasting only a few seconds *(Aaslid R, Lindegaard KF, Sorteberg W, et al. Cerebral autoregulation dynamics in humans. Stroke 1989; 20: 45–52.; Tiecks FP, Lam AM, Aaslid R, et al. Comparison of static and dynamic cerebral autoregulation measurements. Stroke 1995; 26: 1014–1019.)* We followed the White Paper, the CA-focused guideline and the recommendation #3 (Revised) 'Recordings of spontaneous fluctuations of arterial blood pressure and cerebral blood flow for transfer function analysis should last a minimum of 5 min, assuming stationary physiological conditions and uninterrupted good quality data' *(Panerai RB, Brassard P, Burma JS, et al. Transfer function analysis of dynamic cerebral autoregulation: a CARNet white paper 2022 update. J Cereb Blood Flow Metab. 2023;43(1):3–25).* The Autoregulation Index (ARI) will be the parameter adopted to express dynamic CA, with impaired dynamic CA being defined as an ARI < 4.**

**Our protocol is as follows: assessment of the baseline (T0) for 5 minutes, a target of 65 mmHg for 5 minutes (T1). Next, a target of 75 mmHg for 5 minutes (T2) and a target of 85 mmHg for 5 minutes. The pressure will not remain fixed; it will be around the established targets.**

**In the original manuscript we did not describe the different sites for measuring invasive blood pressure. We have added this information now, thank you for your suggestion.**

**Q8**. Please clarify the endpoint ‘Brain dysfunction’ for the 7- and 28-day follow up.

**Replay –** **The definition of brain dysfunction that we will employ is as follows: a Glasgow Coma Scale score of less than 15 or the presence of disorientation, disorganized thinking, or agitation as reported by the attending physician. This definition applies irrespective of sedative/analgesic use and in the absence of neurological diseases (such as dementia, cerebrovascular disease, brain tumors, or previous traumatic brain injury). We have adopted this definition, which was used by Crippa et al (Crippa, I.A., Subirà, C., Vincent, JL. *et al.* Impaired cerebral autoregulation is associated with brain dysfunction in patients with sepsis. *Crit Care* 22, 327 (2018)) and is based on the definition of sepsis-associated encephalopathy.**

**Data from medical records will be used to access such information. Any change in the level of consciousness or the use of antipsychotics or dexmedetomidine will be considered indicative of brain dysfunction.**

**The decision to choose two time points is based on the rationale that analyzing only on the 28th day or at discharge might be influenced by unmeasured confounding factors, so the seventh day may provide a more accurate assessment.**

**Q9.** It appears that CNS infections are not excluded (cf Table 1), is this correct? Please clarify.

**Reply- This is an important point. We are not including any patient with any neurological injury and this includes central nervous system infection.**

Q10. The sample size is not supported by data on statistical dispersion, any minimal differences to be detected etc. No consideration of study attrition is given (for example poor TCD windows). The sample size statement as written is not adequate.

**Reply – Thank you. We are estimating a 1-point difference in ARI between the different MAP targets, while maintaining the 95% confidence interval. Therefore, we need a sample of 45 patients. These data can also be seen in the following publication: Fiona G. Brodie, Emily R. Atkins, Thompson G. Robinson, Ronney B. Panerai; Reliability of dynamic cerebral autoregulation measurements using spontaneous fluctuations in blood pressure. Clin Sci (London) 1 March 2009; 116(6):513–520.**

Q11. The recruitment statement does not seem conducive to consecutive enrollment but a pragmatic, logistic approach. Please explain and clarify (a pragmatic, convenience approach would be acceptable for a pilot trial).

**Reply - All patients that start noradrenaline infusion at the São Rafael Hospital are transferred or are already in intensive care units. Every day, the team intensivists in charge, will report to the researcher team those patients who require new vasopressors, and the researchers will actively search the units as well. If clinical suspicion is of septic shock, the individual will enter the study's current flow, adhering to eligibility criteria. If another hypothesis is defined in the subsequent 48 hours, the patient will be excluded. Each day of the week there will be a researcher available to collect data and interview the patient or their family. Individuals can be recruited within 48 hours of the initiation of vasopressors, making data collection feasible.**

Q12. Please elaborate on study blinding. It appears the investigators are the clinical staff involved in patient managementand will also perform the subsequent analyses? Sequential numbering in such a small sample size does not guarantee blinding. Please explain and clarify (incomplete blinding would be acceptable in a pilot trial).

**Reply: Two researchers are involved in the data collection. During data collection, they have access to Transcranial Doppler parameters, such as mean velocity and pulsatility index. However, ARI from Transfer Function Analysis, CrCP, and compliance parameters for non-invasive ICP are needed for further analysis.**

**The pilot study is not blind. at the time of data collection, it will be impossible to know the value of the ARI or CrCP. Moreover, during data analysis, when runnint the software, the classification of the patient could be blind to the operator.**

Q13. Please elaborate on how sedatives and analgesic use will be adjusted for. The statement ‘GCS<15… regardless of sedation’ appears counterintuitive for the study aims?

**Reply: This term ‘GCS<15… regardless of sedation’ was used for brain dysfunction (BD). Independent, will be describe like BD if GCS < 15 (in the absence of neurologic disorders) or if disorientation, disorganized thinking or agitation were reported by the attending physician, regardless of sedative/analgesic. We don’t pretend to be adjusted for this variable because if the patient exhibits agitation despite the use of sedatives and analgesics, the data will be recorded in the same way as for those who were not using them.**

**The primary objective is to evaluate cerebral dysfunction associated with sepsis without making a distinction between sedated and non-sedated individuals. Nonetheless, a secondary analysis can be conducted to ascertain if sedated patients present a similar profile of brain dysfunction.**

Q14 Please elaborate on procedures to adjust for adjunct therapies, importantly ventilatory support since likely to influence haemodynamic variables. While blood pressure, and hence CPP, is the primary variable of interest, how will for example differences in cardiac output, arterial CO2 and O2 be managed since they all affect autoregulation and compliance.

**Reply: Thank you for raising this very relevant point. As we are aware of the significant influence of CO2 and O2 levels on cerebral hemodynamics, all patients are required to have an arterial catheter. Blood gas analysis will be conducted both before and after the protocol to observe the variability of gases in each patient. For mechanically ventilated individuals, capnography will be employed, and the value will be recorded at the beginning of each blood pressure target.**

**Regarding cardiac dysfunction, we will describe the presence or absence of cardiac dysfunction, dichotomized based on the left ventricular ejection fraction. We will utilize the patient's most recent echocardiogram for this purpose (performed during the same hospitalization or prior). We will also describe the number of patients with severe diastolic dysfunction.**

Q15. Please explain and support why MAP levels are not randomised but always done in the same order (if this is indeed correct). While the repeated measures ANOVA is appropriate, what post hoc testing will be performed? Will any methods be used to assess non-linear correlations? Will multivariable regressions be performed? Including adjusting for confounders / co-variates?

**Reply- We initially did not anticipate that randomizing the MAP levels would alter the autoregulation indices. Our rationale was based on the distinct time intervals required to attain individual blood pressure targets, thus assuming that preceding blood pressure levels would not exert influence on the assessed target.**

**Changes in cerebral hemodynamic (ie: ARI/ CrCP, P1P2 ) at T1, T2 and T3 will be assessed with repeated measures ANOVA.**

**Furthermore, logistic regression with both categorical and continuous independent variables will be used to build predictive models for the occurrence of impaired cerebral autoregulation and brain dysfunction. The binary classification (presence + vs. absent) will be used as the outcome variable. Logistic regression models for the presence or absence of impaired cerebral autoregulation and brain dysfunction will be constructed using those variables with statistically significant differences between groups.**

Q16. How will ‘missing and incorrect data’ be managed (since no imputations will be made)? This again highlights the need to explain measures in place against study attrition.

**Reply: Thank you. We will modify and impute the missing data by the sample mean.**

**Q17 MINOR.** A thorough linguistic revision for style, syntax and grammar would improve the manuscript.

**Reply - Thank you, we will do a linguistic review.**

**Reviewer #2**

Q18. I would recommend to improve the writing quality before publishing. For instance, Figure 1 contains multiple errors: MAP targets during the intervention protocol are false; please addhours to “< 48 ICU admission”; cererbral artery : remove the extra R.

**Reply: Thank you for your careful reading of the manuscript. We have corrected these mistakes.**

Q.19. Some sentences must be rephrased. As an exemple: “All the patients that use vasoactive drugs will be considered to enroll, so the criteria for inclusion and exclusion will be applied (Table-1 )”

Reply: **We have corrected this table legend.**

Q.20 There is no major methodological issue with the study protocol. Here are listed minor concerns:- COPD is presented as an exclusion criterion. It is a frequent condition in critically ill patient. Could you limit this exclusion criterion to COPD patients with severe hypercapnia?

**Reply: We agree. The understanding that carbon dioxide (CO2), as the principal metabolic factor, plays a substantial role in influencing cerebral hemodynamics is well-established. Hypercapnia, in particular, has the potential to adversely impact cerebral autoregulation (The effect of hypercapnia on the directional sensitivity of dynamic cerebral autoregulation and the influence of age and sex. Panerai RB, J Cereb Blood Flow Metab, 2023). We will exclude COPD with hypercapnia.**

Q21. Section “Who will take informed consent?”: according to inclusion criteria, many patients could receive information and consent. The paragraph only refers to legal representatives.

**Reply: Informed consent will be obtained from the patient or their legal representative. Please, see line 162.**

**Q22.** Section “Methods: data collection and management Please collect use of sedation and/or neuromuscular blocking agents since it could affect cerebral hemodynamicand brain dysfunction.

**Reply: Details will be provided regarding the administration of sedation, the specific drug utilized, and the use of neuromuscular blocking agents.**

Q23. Please collect D28 mortality. It could be interesting to analyze the association between cerebral hemodynamic and mortality.

**Reply: Thank you very much for this valuable insight. We have now incorporated the assessment of the association between cerebral hemodynamics and mortality on Day 28 ed version of the manuscript**

Q24. Evaluation of brain dysfunction could be standardized, using specific scores (CAM-ICU for instance)

**Reply: Thank you for your suggestion, we also will collect the CAM ICU.**

Q25. PLOS authors have the option to publish the peer review history of their article (what does this mean?). If published, this will include your full peer review and any attached files.If you choose “no”, your identity will remain anonymous but your review may still be made public.

**Reply – YES**
