# Supplementary material for: Impact of different blood pressure targets on cerebral hemodynamics in septic shock: A prospective pilot study protocol—SEPSIS-BRAIN
Source: PLoS One. 2024 Oct 14;19(10):e0304412. doi: 10.1371/journal.pone.0304412 (PMC11472940; doi:10.1371/journal.pone.0304412)
Supplement: Attachment — Submitted filename: RESPONSE_TO_REVIEWERS.docx [file pone.0304412.s006.docx]

Dear Dr. Vincenzo Lionetti,

Thank you very much for conducting the review process for our manuscript PONE-D-23-22748R1 and for the opportunity to address the points raised by the reviewers. We found their comments relevant and these were taken into account to produce a revised version of the manuscript. We trust that you will find that our manuscript has been considerably improved, and we hope the revised version will meet your acceptance criteria. Our replies to each of the comments from the reviewers are given below where we also explain corresponding changes made to the manuscript.

**Comments to the Author:**

**Q2. Is the protocol technically sound and planned in a manner that will lead to a meaningful outcome and allow testing the stated hypotheses?**

**Comment:** The manuscript should describe the methods in sufficient detail to prevent undisclosed flexibility in the experimental procedure or analysis pipeline, including sufficient outcome-neutral conditions (e.g. necessary controls, absence of floor or ceiling effects) to test the proposed hypotheses and a statistical power analysis where applicable. As there may be aspects of the methodology and analysis which can only be refined once the work is undertaken, authors should outline potential assumptions and explicitly describe what aspects of the proposed analyses, if any, are exploratory.

**Replay:** Thank you for the clarifications. We have made some adjustments and highlighted key points for clarity. Our protocol is a pilot project aimed at evaluating physiological data. In this context, we realized the need to refine our primary objective.

Our primary outcome is to characterize cerebral hemodynamics in patients with septic shock across different blood pressure targets. This characterization will be conducted using Transcranial Doppler and B4C. The ARI, CrCp and RAP will be assessed by transcranial Doppler RAP, while the P2/P1 ratio by B4C device.

**Q3.** **Is the methodology feasible and described in sufficient detail to allow the work to be replicable?**

**Comment:** Descriptions of methods and materials in the protocol should be reported in sufficient detail for another researcher to reproduce all experiments and analyses. The protocol should describe the appropriate controls, sample size calculations, and replication needed to ensure that the data are robust and reproducible.

**Reply:** Thank you for your thoroughness in describing the methodology. We have provided detailed information on patient selection, sample collection location, duration, intervention procedures, and data collection. Additionally, we have included extensive details on measurements of cerebral hemodynamics, which have been well-documented in the literature, including critical care patients *(Panerai, RB J, Cereb Blood Flow Metab, 2023; Caldas J, Annals of Intensive Care Medicine 2019; Caldas J, Shock, 2019; Bergman L, Am J Obstet Gynecol. 2022; Salinet AS, J Cereb Blood Flow Metab. 2019; Simpson DM, J Cereb Blood Flow Metab. 2022; Caldas J, Neurophysiology, 2019;)* .Additionally, we have included details to enhance reproducibility, such as the sequence for achieving mean arterial pressure targets. We will not include control groups in our study, similar to the approach taken by others researchers (Crippa et al. Critical Care 2018; Pierrakos et al. Annals of Intensive Care 2013,). Our study is meticulously crafted to elucidate physiological parameters within a pilot investigation. Its principal objective is to characterize the pattern of cerebral hemodynamics within this specific population. This pilot initiative aims to provide foundational data for future studies, promoting adherence to the same protocol and facilitating the utilization of amassed data for sample size calculations.

**Q5 Is the manuscript presented in an intelligible fashion and written in standard English?**

Reply – Thank you, we have corrected English errors.

**Review Comments to the Author**

**# Define the primary outcome or outcomes clearly and with specificity.**

**Comment**: First, the authors must be clear when they specify the primary outcome. As it currently stands, the authors state that the primary outcome is "changes in cerebral hemodynamic variables and other parameters''. This is non-specific.This is not clarified in other areas in the manuscript. The sample size is calculated against ARI, so I surmise that this is one of the primary outcomes. From Figure 1, I further surmise that ARI is one of three measurements from TCD, the other two being CrCP and RAP. Only one measurement of interest is taken from B4C, which is P2/P1. At no time are these four measurements, taken together, identified as the "cerebral hemodynamics'' to which the authors refer. This is inadequate. The authors need to be clear about which of the measurements being taken are used as the primary outcome or outcomes.

**Replay:** Thank you for the feedback; I'm glad to hear that the comments were helpful in refining our project. We have made adjustments accordingly.

Our primary objective now focuses on evaluating cerebral hemodynamics across different blood pressure targets. It's important to note that all components of cerebral hemodynamics will constitute the primary outcome. This study serves as a pilot project, and the data obtained will be instrumental in guiding future research focusing on specific indices as primary objectives.

Additionally, we have included the 65 mmHg blood pressure target as a secondary objective in our study. This is in line with current guidelines and will contribute to the overall description of cerebral hemodynamics.

**# The intervention is unclear**

**Comment:** The authors state that the interventions being applied are three different MAP targets, yet they do not describe how these targets will be achieved. This is a major omission.

.**Replay:** Thank you for bringing attention to this point. I was thinking of describing it as follows: The primary blood pressure target will be the mean arterial pressure (MAP) level of the patient at the time of collection, with subsequent targets invariably following the order of 65 > 75 > 85 to ensure consistency in the collection pattern.

**# The sample size statements are irreplaceable.**

**Comment:** A previous reviewer noted that the original manuscript's description of the sample size calculation was inadequate. I'm sorry to say that the authors' response is inadequate. The calculation of the sample size is important and the revisions do not allow for confirmation of the size estimates.

The authors cite a separate article. This is inappropriate. The current manuscript must stand alone. The authors must identify which pieces of information they took from the reference and show how they arrived at the current sample size. In particular, they must justify the following:

(1) that a 1-point increase in ARI is a clinically relevant change, and

(2) the variability (expressed as a standard deviation or variance) around the ARI readings at the target MAP of 65.

**Replay:** Thank you for your comment. We cited the article separately to highlight the observed differences in pressure between groups as an example. Our study is indeed a pilot project, with a sample size we deemed feasible to collect within this timeframe. The physiological data obtained from this study are intended to serve as a foundation for subsequent research endeavors. Our aim is to facilitate the development of larger studies that can follow this protocol and derive a correct sample size based on the generated data.

**# Linear regression may be inappropriate**

**Comment:** The authors state that "Linear regression can be used to formulate a model based on different pressure targets." This may or may not be appropriate depending on the way that the model is operationalised. At present there is not enough information to tell whether this is appropriate because the statement is inadequately specified.

The authors must use a regression model that accounts for the non-independence of measurements. That is, each patient serves as his or her control. The authors cannot simply ignore this major feature of their data since independence of measurements is one of the assumptions of linear regression and it is voided by the authors' study design. The authors must be clear about how they plan to account for this.

The authors may need to seek the assistance of a professional biostatistician to refine their methods.

**Replay:**I appreciate your input. Our intention is to be as transparent as possible.Changes in cerebral haemodynamics indexes at different times, T0, T1, T2 and T3 will be assessed with repeated measures ANOVA. Additionally, in the chapter that deals with the statistical model, we will add the fact that the measurements will be taken repeatedly and that there will be interdependence, and therefore, the most suitable model is mixed-effects, nonlinear regression models will be tested.
