# Supplementary material for: Impact of different blood pressure targets on cerebral hemodynamics in septic shock: A prospective pilot study protocol—SEPSIS-BRAIN
Source: PLoS One. 2024 Oct 14;19(10):e0304412. doi: 10.1371/journal.pone.0304412 (PMC11472940; doi:10.1371/journal.pone.0304412)
Supplement: Attachment — Submitted filename: Response to Reviewers.docx [file pone.0304412.s007.docx]

Dear Vincenzo Lionetti,

Thank you very much for conducting the review process for our manuscript PONE-D-23-22748R1 and for the opportunity to address the points raised by the reviewers. We found their comments relevant and these were taken into account to produce a revised version of the manuscript. We trust that you will find that our manuscript has been considerably improved, and we hope the revised version will meet your acceptance criteria. Our replies to each of the comments from the reviewers are given below where we also explain corresponding changes made to the manuscript.

**Comments to the Author:**

**Q5 Is the manuscript presented in an intelligible fashion and written in standard English?**

Reply – Thank you, we've done an extensive review, we hope it's satisfactory.
